# Electromechanical Impedance Sensing Under Humid Conditions: Experimental Insights and Compensation Using Machine Learning

**DOI:** 10.3390/s26030943

**Published:** 2026-02-02

**Authors:** Mads Kofod Dahl, Jaamac Hassan Hire, Milad Zamani, Alexandru Luca, Farshad Moradi

**Affiliations:** 1 Department of Electrical and Computer Engineering, Aarhus University, 8200 Aarhus, Denmark; 2 SDU Microelectronics, Department of Mechanical and Electrical Engineering, University of Southern Denmark, Campusvej 55, 5230 Odense, Denmark; 3 Department of Food Science, Aarhus University, Agro Food Park 48, 8200 Aarhus, Denmark

**Keywords:** SHM, EMI, electro mechanical impedance, structural health monitoring, humidity, reinforced concrete, piezoelectric, multi modal sensing, machine learning, CNN

## Abstract

This work investigates the effect of ambient humidity on the Electromechanical Impedance (EMI) signatures of steel-reinforced concrete (RC) for structural health monitoring (SHM). The influence of varying relative humidity (%RH) is quantified using three RC blocks containing piezoelectric sensors bonded to the steel reinforcements of the RC blocks. We show that the the Root Mean Squared Deviation (RMSD) score is strongly affected by humidity, highlighting the need to address humidity effects to achieve robust damage detection using EMI. Using the reactive component of the EMI (*X*) in the range of 20 kHz and 120 kHz, a three-layer one-dimensional convolution neural network (1D-CNN) was able to estimate ambient %RH between 20% and 80%, with a Mean Absolute Error (MAE) of 2.14%RH. The results highlight the significant impact of humidity on EMI-based SHM and suggests that the imaginary part of the EMI signature can be used to detect the effect of humidity. This work provides a foundation for more robust SHM systems in humidity-varying environments applicable to a wide range of concrete infrastructure.

## 1. Introduction

The Electromechanical Impedance (EMI) technique enhances traditional potential-monitoring-based structural health monitoring (SHM) methods by addressing limitations such as inaccuracy, non-continuous monitoring, incompatibility with wireless sensor networks (WSN), and the need for expert data analysis. One area where SHM is both critically important and challenging to implement is in sea bridges. The humid and salty atmosphere accelerates corrosion in steel reinforcement, which can lead to costly and potentially catastrophic structural failures. Early and accurate corrosion detection is therefore essential for maintaining the integrity of such infrastructure.

However, existing electro-potential-based corrosion sensors lack precision and continuous monitoring capabilities. Several studies have demonstrated that the EMI technique can provide earlier and more accurate detection of corrosion compared to conventional methods [1,2,3]. Within the broader context of smart structures with self-sensing capabilities, much work has been conducted using piezoelectric sensors [4], yet embedded EMI-based approaches remain comparatively understudied. Despite its advantages, EMI-based SHM still faces challenges that limit its broader adoption.

A key challenge is the sensitivity of EMI measurements to changing ambient conditions such as temperature and humidity [3]. Temperature effects have been widely studied, and multiple mitigation approaches have been proposed [5,6,7]. Our previous work also quantified the temperature dependence of EMI signatures and explored machine learning (ML) methods to compensate for these variations [8].

In contrast, the effect of humidity has received far less attention. Previous studies have shown that changes in humidity shift both the frequency and amplitude of resonant peaks [9,10], which complicates the use of the most commonly used Root Mean Squared Deviation (RMSD) damage detection algorithm. Tong et al. [10] further observed that shifts in resonant peaks of the real part of the impedance vary across sensors, even under identical humidity levels, showing the short comings of using the frequency and amplitude shifts of the real part to extract humidity information.

While ML has been applied to EMI data for damage detection and temperature compensation [11,12], to our knowledge, no works have used ML to measure humidity. Moreover, most prior works use surface-mounted piezoelectric transducers, which detect cracking or surface degradation. In this work, we embed the transducer within the concrete, by bonding it to an embedded steel sensor probe, and use 3D-printed covers to protect the transducer. Having the probe inside the concrete allows for detection of corrosion at an earlier stage, before visible cracking occurs.

In this work, we analyze the EMI signatures of three reinforced concrete (RC) blocks containing embedded piezoelectric sensors bonded to steel rods cast within the specimens. The samples are placed in a humidity chamber, and the relative humidity (%RH) is varied from 20% to 80%. Using the resulting data, we quantify how humidity affects the EMI response of RC and compare ML models trained on different features to find the feature best suited for the detection of relative humidity.

This work provides an experimental investigation of the influence of ambient humidity on the EMI technique, demonstrating that robust SHM based on EMI must account for humidity fluctuations. Using the collected experimental data, we show that different impedance representations and frequency ranges exhibit different sensitivities to humidity. In particular, we demonstrate that the real part of the impedance is a poor indicator of humidity, whereas the imaginary part shows significantly stronger predictive capability. This conclusion is supported by the results of an ML prediction. Overall, this work highlights humidity as a critical challenge for EMI-based SHM and suggests practical considerations as well as a path forward through the development and deployment of humidity compensation schemes using machine learning models trained on the imaginary component of the impedance.

The structure of the paper is as follows: Section 1 is a theoretical exploration using Liang’s model [13], illustrating the effect of humidity on the EMI technique. Section 2 describes the samples, humidity chamber setup, and ML model training. Section 3 analyzes the humidity effect and evaluates the ML model’s performance. Section 4 details the findings and limitations. Section 5 contains our conclusions.

### The EMI Technique and the Effect of Humidity

The Electromechanical Impedance (EMI) technique uses a piezoelectric sensor, typically a lead zirconate titanate (PZT) element, bonded to the Device Under Test (DUT). The EMI signature is extracted over a broad frequency range, usually in the kilohertz domain. Generally, only the real part of the impedance (*R*) is analyzed in order to assess the condition of the DUT [6,14].

Damage detection is performed by comparing the real part of the EMI signature in a healthy state with that in a potentially damaged state, using a metric such as the Root Mean Squared Deviation (RMSD). The RMSD quantifies deviations between reference and subsequent measurements, with resonant peaks in the real part showing the highest sensitivity to structural changes [3].

(1) $$\mathrm{RMSD}=\sqrt{\frac{\sum_{i=1}^{N}{\left(R_{D}\left(i\right)-R_{\mathrm{Ref}}\left(i\right)\right)}^{2}}{\sum_{i=1}^{N}{\left(R_{\mathrm{Ref}}\left(i\right)\right)}^{2}}}$$

where *i* denotes the *i*-th datapoint of the EMI signature and *N* is the total number of datapoints. $R_{Ref}$ is the EMI signature of the DUT in the reference state, and $R_{D}$ represents the EMI signature in a subsequent measurement.

The analytical model proposed by Liang et al. [13] relates the mechanical impedance of the PZT patch ($Z_{T}\left(\omega \right)$) and that of the host structure ($Z_{S}\left(\omega \right)$) to the complex electrical admittance ($\bar{Y}$) at the PZT terminals:

(2) $$\begin{array}{cc} \bar{Y} & =\omega j\frac{wl}{h}\left[\left(\bar{\varepsilon_{33}^{T}}-d_{31}^{2}\bar{Y^{E}}\right)\right.\\ \; & \left.+\left(\frac{Z_{T}}{Z_{S}+Z_{T}}\right)d_{31}^{2}\bar{Y^{E}}\left(\frac{\tan(\kappa l)}{\kappa l}\right)\right] \end{array}$$

The corresponding electrical impedance is given by $Z=1/\bar{Y}$, where *w*, *h*, and *l* are the width, height, and length of the PZT patch. $\varepsilon_{33}^{T}$ denotes the complex electric permittivity of the PZT, $d_{31}$ the piezoelectric strain coefficient, $Y^{E}$ the complex Young’s modulus, and κ the one-dimensional wave number associated with the angular frequency ω. This relationship links the EMI signature to the mechanical and electrical properties of both the DUT and the PZT patch through $Z_{S}\left(\omega \right)$ and $Z_{T}\left(\omega \right)$. Because the mechanical impedance of the PZT is much smaller than that of the DUT, variations in the electrical impedance mainly reflect changes in the mechanical impedance of the DUT, providing a basis for detecting structural damage. The impedance of the host structure can be described by the following:

(3) $$Z_{S}=c+mj\omega +\frac{k}{j\omega }$$

where *m*, *k*, and *c* represent mass, stiffness, and damping, respectively.

Humidity alters these parameters through its effect on both the concrete matrix and the PZT element. Concrete is a highly porous material subject to capillary suction, through which liquid water and water vapor are absorbed and transported into its pore network. The added moisture changes the material’s mechanical and electrical characteristics. It is clear that as water is absorbed, the mass of the concrete will increase. Bordas et al. [15] and Abbas et al. [16] reported that increasing moisture decreases electrical resistivity, while Wang et al. [17] showed that wet concrete exhibits a higher stiffness compared to dry concrete. There is debate within the literature on whether humidity increases or decreases damping, but for the purposes of this work, we will assume that an increase in humidity leads to an increase in damping.

**Figure 1:**
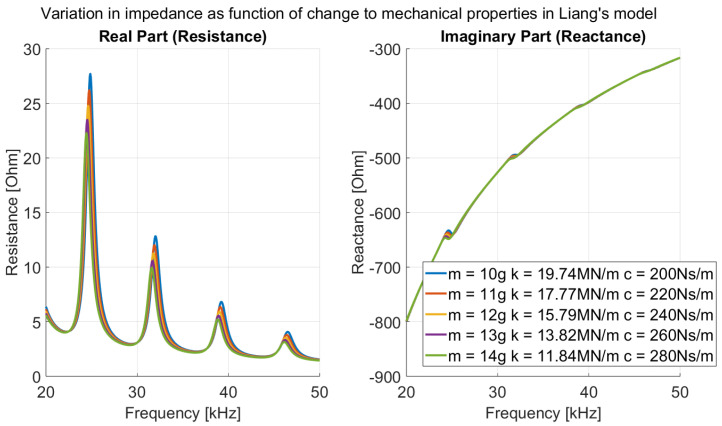
Effect of mechanical properties on impedance ($Z_{S}$) in Liang’s model.

In the context of Liang’s model, an increase in humidity leads to an increase in the effective mass, stiffness and damping of the host structure. The combined influence of increased *m*, *k* and *c* is illustrated in Figure 1, showing a reduction in amplitude and a slight downward frequency shift in the real part, with minimal change in the imaginary part.

In the experiments, we observed a large change in the imaginary part of the impedance caused by humidity that was not present in the simulated system seen in Figure 1. This discrepancy is solved when considering how humidity also affects the electrical properties of the system.

**Figure 2:**
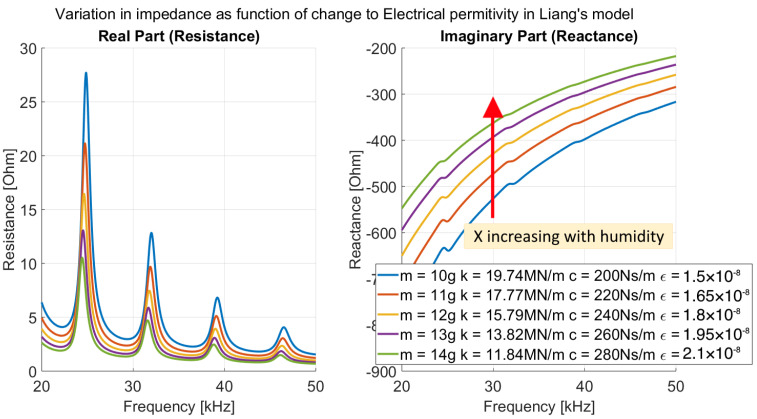
Effects of mechanical properties and electrical permittivity on impedance ($Z_{S}$) in Liang’s model.

The permittivity of water ($\epsilon_{\mathrm{Water}}\approx 80$) is much higher than that of concrete ($\epsilon_{\mathrm{Concrete}}\leq 10$ [18]). As water fills the pores of the concrete, the effective permittivity increases, altering the electrical response of the system. When this increase in electrical permittivity is incorporated alongside the mechanical effects, the simulated results better align with experimental observations, as shown in Figure 2.

These combined phenomena make humidity a critical variable in EMI-based structural health monitoring. Natural fluctuations in environmental humidity—both indoors and outdoors—can cause changes in the EMI signatures that may be misinterpreted as structural damage. Accounting for these effects is therefore essential for accurate, long-term SHM in reinforced concrete structures.

## 2. Methods

In this section, we outline the methodology and experimental setup employed to obtain the EMI and humidity data set, as well as the training of the ML model.

### 2.1. Samples

**Figure 3:**
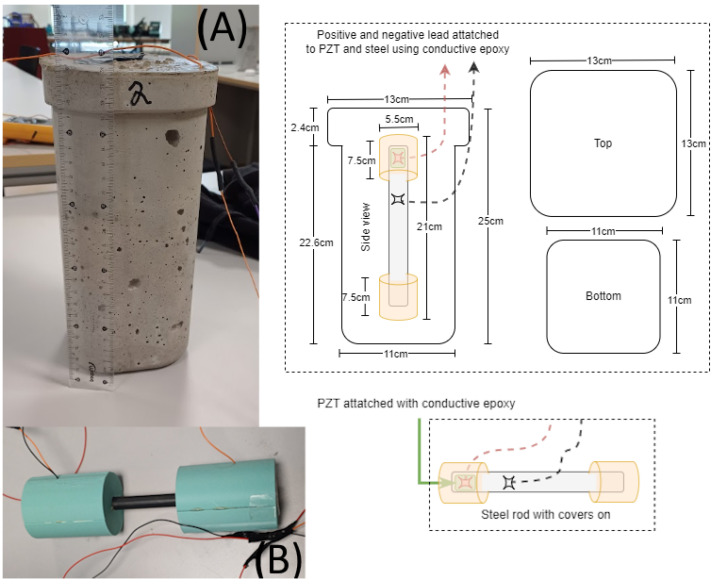
(**A**) The concrete sample with the steel rod shown on (**B**) embedded inside of it. The steel rods use 3D printed covers for protection of the PZT. The red and black dashed lines indicating the positive and negative lead respectively.

In total, three concrete blocks were fabricated for the experiments. Each block contained a 316 stainless steel rod with an attached PZT patch, bonded using a conductive epoxy, and hollow 3D-printed end covers to limit vibration attenuation from the surrounding concrete, as shown in Figure 3. The PZT transducers were 10 mm × 10 mm × 1.5 mm APC-840 patches (American Piezo), bonded with CW2400 conductive epoxy (Chemtronics), which was cured at room temperature for 72 h. The rods were encased in fast-setting concrete from Skalflex, mixed at a water-to-cement ratio of approximately 1:11, and cured in the laboratory for 28 days.

### 2.2. Test Setup—Data Collection Experiment

The purpose of the test was to create a high-quality data set that could be used to accurately investigate the correlation between humidity and impedance.

**Figure 4:**
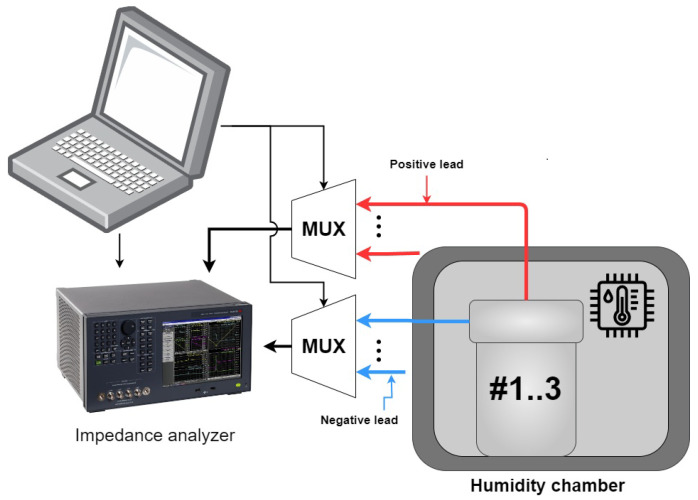
Samples #1 through #3 were placed in the humidity chamber with, two multiplexers being used to switch between the samples the impedance analyzer was connected to.

A diagram of the test setup can be seen in Figure 4; an impedance analyzer (Keysight E4990A) was used to obtain the impedance data of 3 different samples, a multiplexer was used to switch between the samples, and a laptop was used to control the impedance analyzer and multiplexer. An EMI data sweep was performed every third hour at a 10 Hz resolution over the frequency span of 20–400 kHz.

**Figure 5:**
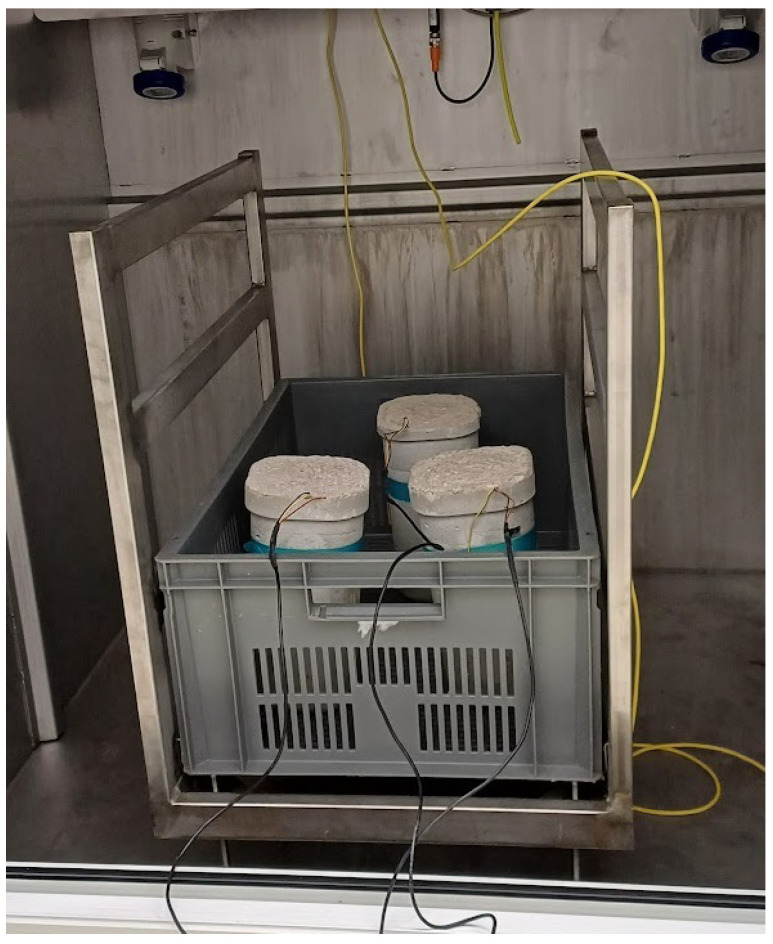
Photograph from the data collection experiment: the 3 samples in the humidity chamber with wires leading to outside the impedance analyzer.

Before the test was initiated, the samples were dried for 4 days in a 0 %RH chamber at 55 °C. The samples were then moved to a custom-made stainless steel 1 m^3^ chamber (as seen in Figure 5) where the RH was adjusted from 20 to 80 % with steps of 20 %RH over 4 weeks using a CA storage control system (Storex b.v., Gravendeel, The Netherlands). The chamber was indirectly cooled and heated by air from a walk-in storage room maintained at a mean temperature of 25.24 °C with a standard deviation of 0.37 °C over the duration of the experiment. Because the observed temperature variation was minimal, the effect of temperature variance was assumed to have a negligible effect and excluded from subsequent analysis.

The RH in the chamber was increased approximately once per week when the impedance measurements where stable; this was carried out to ensure the concrete had reached full saturation at the target RH level. The RH in the chamber was monitored using an nSens-HT-ENS T/RH sensor (Novasina AG, Lachen, Switzerland) and controlled by adding a container of distilled water and injecting dry air as needed. The temperature and humidity data from the sensor were automatically recorded every 5 min and stored using the Storex Autostore Data Manager Cloud Plus system (Storex B.V., Gravendeel, The Netherlands).

### 2.3. Training the ML Model

To ensure the reliable performance of the machine learning model, we make a key assumption: the absorption of water vapor into the concrete is linear over the duration of the experiment. Consequently, instead of using the air humidity reported by the environmental chamber as the target, we assume that the internal humidity of the concrete increases linearly from 20% to 80% over the duration of the test. On day #60, a physical connection on Sample 1 became loose, on day #79, the samples were taken out of the chamber and the problem was corrected. Moving the samples caused a dip in the RMSD score. For this reason, data collected between day#60 and day#90 were excluded from the analysis.

The dataset was randomly split into 80% training and 20% validation subsets using the random_split() function from PyTorch [19]. All models share the same network topology, consisting of a three-layer one-dimensional convolutional neural network (1D-CNN) followed by a fully connected regression head. Each convolutional layer is followed by a ReLU activation function, and global feature aggregation is performed using adaptive average pooling. The fully connected part consists of a hidden layer with ReLU activation and dropout regularization, followed by a single linear output neuron.

Hyperparameter optimization was performed using Optuna [20]. The optimized hyperparameters include the number of convolutional filters, kernel size, stride length, hidden layer dimension, dropout probability, and learning rate. The full hyperparameter search space is summarized in Table 1; wide hyperparameter ranges were used to avoid biasing the model design, allowing Optuna to find suitable configurations for each frequency range and feature. For each experiment, the model configuration with the lowest validation loss was selected.

Models were trained using the Adam optimizer with a batch size of 64 for 300 epochs. This configuration was chosen to ensure stable optimization and convergence across all hyperparameter configurations. In total, 16 models were trained, corresponding to a 4×4 combination of frequency ranges and impedance data representations, as summarized in Table 2.

## 3. Results

From the data collection experiment, we present the complex impedance data over humidity. Furthermore, we compare the RMSD with humidity, highlight the problem of humidity in the EMI signature and show the prediction accuracy of ML models trained on different frequency areas and representations of the impedance data.

### 3.1. Data Collection Experiment

This section presents the results of the humidity data collection experiment, analyzing the EMI in four different representations.

#### 3.1.1. Real Part (*R*)

**Figure 6:**
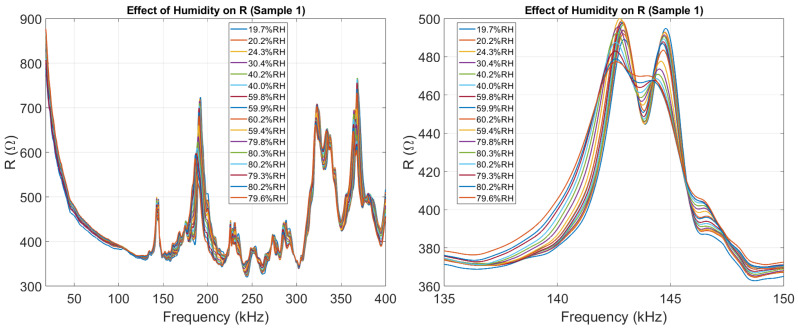
The effect of humidity on the real part of the impedance of Sample 1, highlighting the largest peak appearing between 135 kHz and 150 kHz.

**Figure 7:**
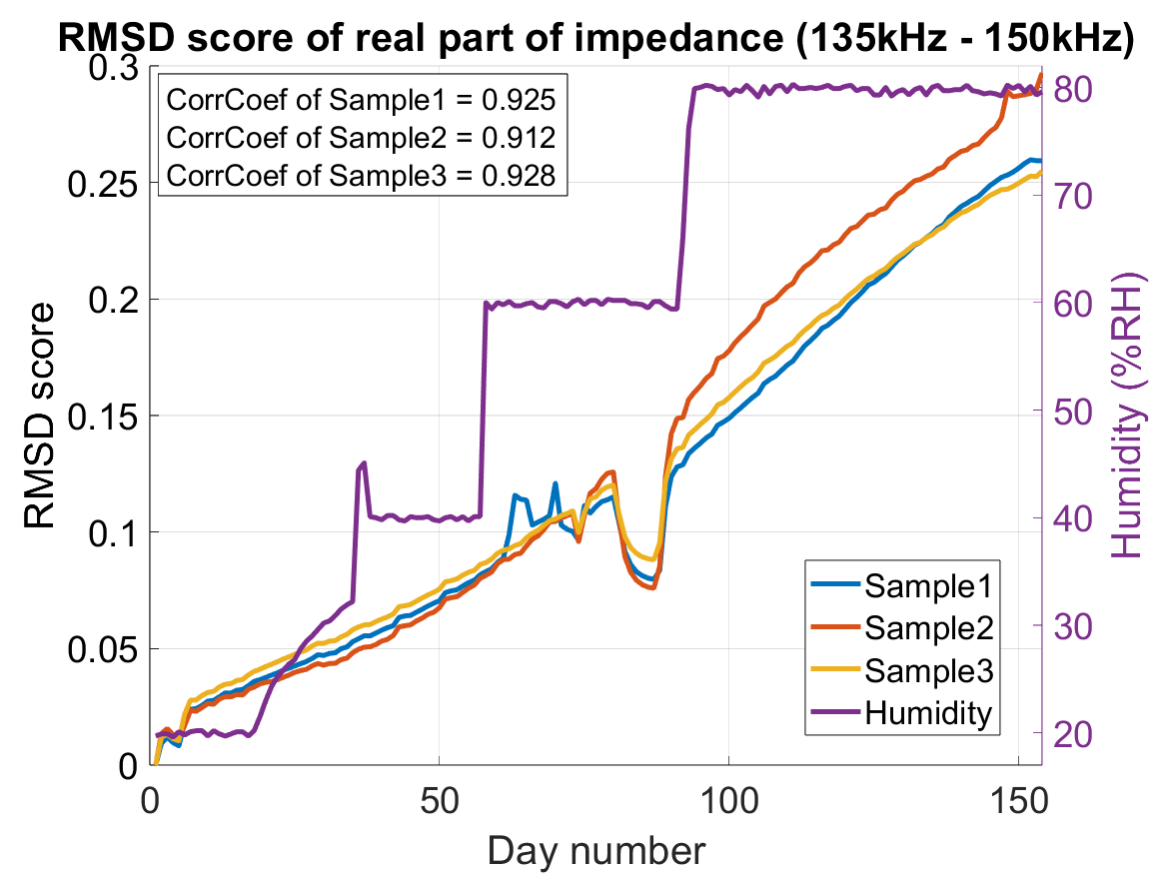
Output of RMSD algorithm applied to *R* in the peak region (135–150 kHz), showing a strong correlation coefficient between RMSD and humidity.

Figure 6 illustrates how humidity affects the real part of the impedance, as discussed in Section The EMI Technique and the Effect of Humidity. The EMI Technique and the Effect of Humidity. The issue is further emphasized in Figure 7, where a damage detection algorithm (RMSD) is applied to the real part of the impedance, demonstrating the practical problems caused by humidity. As humidity varies, the RMSD score—normally an indicator of damage—increases significantly, making it impossible to distinguish between the effects of damage and humidity when analyzing only the real part.

Midway through the experiment, an issue occurred with Sample #1. To correct it, the setup was slightly adjusted, leading to unreliable measurements in all three samples between day#60 and day#90, as shown in Figure 7.

#### 3.1.2. Imaginary Part (*X*)

**Figure 8:**
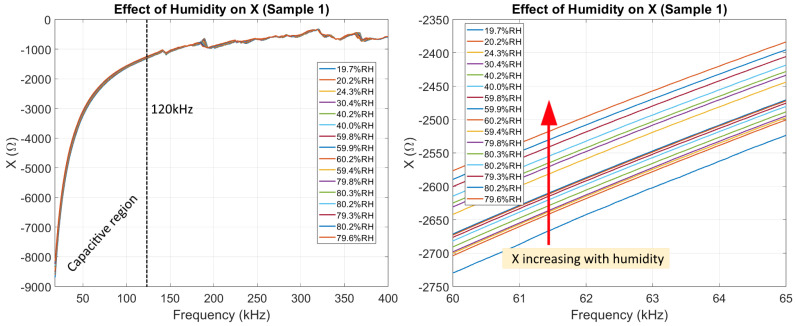
The effect of humidity on the imaginary part of the impedance of Sample 1, highlighting the capacitive region between 20 kHz and 120 kHz. The dashed line at 120 kHz indicates the end of the *Capacitive_region*.

In Figure 8, it can be seen how the imaginary part of Z was affected by humidity. We highlight how no resonant peaks appear below 120 kHz, making the specific effect of humidity on the EMI signature much clearer to analyze below 120 kHz. For this work, we refer to the region with no peaks as the capacitive region of the EMI signature.

#### 3.1.3. Magnitude of Impedance (|Z|)

**Figure 9:**
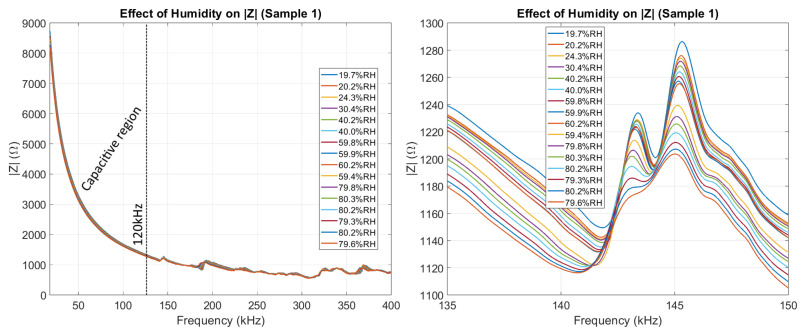
The effect of humidity on the magnitude part of the impedance of Sample 1, highlighting the largest peak appearing between 135 kHz and 150 kHz. The dashed line at 120 kHz indicates the end of the *Capacitive_region*.

In Figure 9, it can be seen how |Z| was affected by humidity. |Z| is dominated by the *X* component of the impedance in the capacitive region (<120 kHz) and dominated by the *R* component of the impedance at higher frequencies (>120 kHz). Because of this, it is expected that the capacitive region of |Z| reacts similarly to the capacitive region of *X*.

#### 3.1.4. Phase of Impedance (θ)

**Figure 10:**
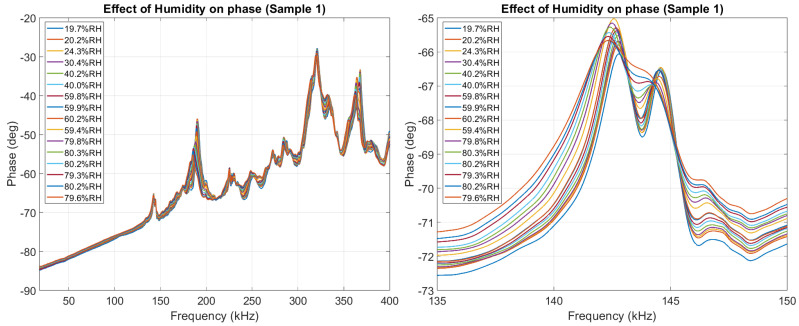
The effect of humidity on the phase of the impedance of Sample 1, highlighting the largest peak appearing between 135 kHz and 150 kHz.

Figure 10 shows how the phase angle θ was affected by humidity. The phase represents the ratio between the reactive and resistive components of the impedance.

#### 3.1.5. ML Model Humidity Prediction

**Figure 11:**
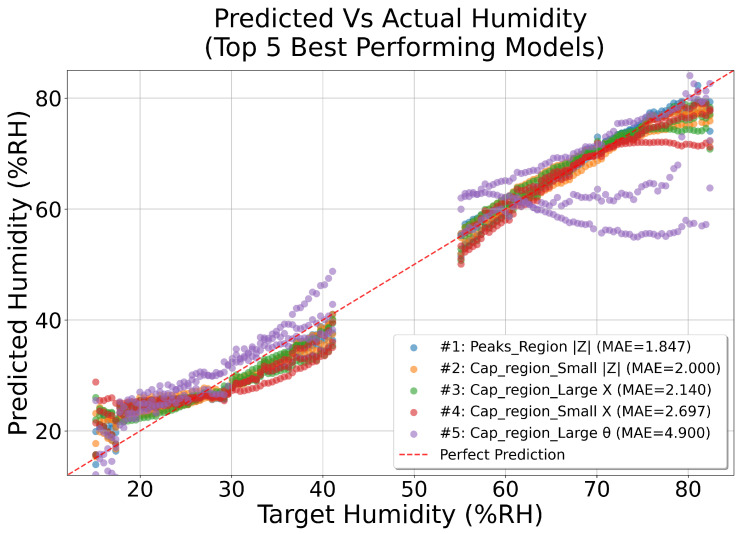
Prediction accuracy of the top 5 preforming models. (The red dashed line indicates what a perfect prediction result would look like).

The prediction results on all frequency ranges and data representations can be seen on Figure 11; the results are also summarized in Table 3. Refer to Table 2 for an explanation of each frequency range and feature.

## 4. Discussion

A key limitation of the study is the small sample size. It is likely that factors such as microstructural variability, surface roughness, or transducer bonding layer influenced the EMI response to humidity. Further work is needed to quantify how these parameters affect the sensitivity of the EMI technique to relative humidity.

The results of using ML, summarized in Table 3, show that using the magnitude of the impedance |Z| in the *Peaks_Region* yielded the lowest MAE. However, because resonant peaks are also affected by structural damage, this region is less suited for humidity estimation in practical applications. Instead, the capacitive region of either the imaginary component *X* or the magnitude |Z| offers a more robust feature space, as it is less sensitive to damage.

Our results indicate that the robustness of the EMI technique can be improved by explicitly considering the imaginary component *X*. In cases where a large RMSD is observed together with large variations in *X*, particularly in the lower frequency range, the response is likely influenced by ambient humidity changes and not only structural damage. Future work should focus on quantifying and generalizing the relationship between humidity and the EMI signature through experiments on a large and diverse set of samples, with the ultimate goal of developing a robust humidity compensation strategy, similar to the existing temperature compensation approaches.

Park et al. [21] showed that debonding alters the capacitance of the patch, thereby modifying the EMI signature in a way that resembles humidity-induced changes. Nonetheless, the temporal characteristics of these two effects differ; humidity may fluctuate in either direction, while debonding progress can only increase. Consequently, long-term monitoring with adequate temporal resolution makes it possible to distinguish between humidity changes and debonding. Additionally, a temporal analysis may increase the reliability of damage detection as, similarly to PZT debonding, damage will only increase.

## 5. Conclusions

Analysis using Liang’s model suggests that humidity influences both the mechanical and electrical properties of reinforced concrete. While changes in the real part of the EMI signature due to humidity can resemble those caused by actual damage, the imaginary part is affected much more strongly by humidity than by damage. This difference makes it possible to distinguish between humidity effects and true structural damage by examining the imaginary component of the EMI signature.

The experimental results obtained in this study follow the same trends predicted by Liang’s model. Increasing humidity produced clear frequency shifts and amplitude reductions in the resonant peaks of the resistive component *R*, while also observing large changes in the capacitive portion of the reactive component *X*. This confirms that humidity has a disproportionately large effect on the imaginary part of the EMI response, consistent with the theoretical predictions.

To further illustrate the risk of misinterpreting humidity as structural damage, the RMSD-based damage detection algorithm was applied to the humidity-varying measurements. As shown in Figure 7, rising humidity leads to elevated RMSD scores, demonstrating that uncompensated RMSD metrics can falsely indicate damage. Additional research is required to quantify this effect and to develop reliable humidity-aware compensation strategies.

Machine learning models can be used to estimate humidity directly from the EMI signature, but their effectiveness depends on selecting regions that are minimally influenced by structural damage. Although the *Peaks_Region* of |Z| yielded the lowest MAE at 1.85%RH, the capacitive regions of |Z| and *X* (with errors of 2.0%RH and 2.14%RH, respectively) are far less affected by damage. Because damage has only a minor influence on the imaginary part in the capacitive range, these regions allow humidity to be isolated from any concurrent damage effects while still relying on the same underlying EMI measurements. This makes the capacitive regions the most suitable choice for robust humidity prediction in real-world conditions. From the results presented in Figure 11, it is clear that the majority of the errors appear at the most dry and most humid ends of the dataset. This is likely a results of assuming a linear absorption curve in the RC where an S-formed absorption curve may be more accurate. Future work should further investigate the nonlinear absorption curves of concrete to improve the accuracy of the predictions.

For practical applications of EMI-based sensors, when deploying the EMI technique in concrete, installing a humidity sensor alongside the EMI probe can be advantageous. Measurements from a conventional humidity sensor can be used to calibrate the EMI response, reducing the large variability observed between similar samples. Depending on various factors, EMI probes are expected to be operational for a significantly longer time than conventional humidity sensors. Once the humidity sensor fails, the calibrated EMI probe can continue to be used to infer humidity with high accuracy, as demonstrated in this work.

1. Despite sample-to-sample variability, relative humidity can be accurately inferred from EMI measurements, confirming a consistent underlying relationship modulated by material-specific factors.
2. The reactive component *X*, particularly in the capacitive region, is the most robust feature for humidity estimation. Future work should expand the dataset to include broader humidity ranges and controlled damage scenarios to further validate this robustness.
3. The findings in this work highlight the significant impact of humidity on EMI-based SHM and offer practical guidance on selecting impedance features that remain dependable under varying environmental and structural conditions.
4. In practical implementations, a traditional humidity sensor can be installed close the the EMI probe, the data from which can then be used to calibrate the EMI signature, increasing robustness and allowing for inference of relative humidity.

## Figures and Tables

**Table 1:** Hyperparameter search space for the 1D CNN model.

Hyperparameter	Search Space	Type
Number of filters ($n_{\mathrm{filters}}$)	{16, 32, 64}	Categorical
Hidden layer dimension (*h*)	{32, 64}	Categorical
Learning rate (η)	$\left[10^{-4},10^{-2}\right]$	Log-uniform
Dropout probability ($p_{\mathrm{drop}}$)	[0.1, 0.5]	Continuous
Kernel size (Conv1) ($k_{1}$)	[3, 30], step 2	Integer
Kernel size (Conv2) ($k_{2}$)	[3, 30], step 2	Integer
Kernel size (Conv3) ($k_{3}$)	[3, 30], step 2	Integer
Stride (Conv1) ($s_{1}$)	[1, 20]	Integer
Stride (Conv2) ($s_{2}$)	[1, 20]	Integer
Stride (Conv3) ($s_{3}$)	[1, 20]	Integer

**Table 2:** Defined frequency ranges for each dataset region and descriptions of the extracted features.

**Region**	**Frequency Range [kHz]**	**Description**
Cap_region_Small	20–35	Narrow band of capacitive region.
Cap_region_Large	20–120	Broad band of capacitive region.
Peaks_Region	135–150	Band containing a major peak in all 3 datasets.
Full	20–400	Full frequency sweep covering all measured data.
**Feature**	**Definition**	**Description**
Impedance	Z=R+jX	Complex representation of EMI
Re (Real part)	R=Re(Z)	Resistive component
Im (Imaginary part)	X=Im(Z)	Reactive component
Mag (Magnitude)	$\left|Z\right|=\sqrt{R^{2}+X^{2}}$	Absolute value of impedance
Phase (Angle°)	$\theta =\tan^{-1}\;\left(\frac{X}{R}\right)\frac{180}{\pi }$	Phase angle in degrees

**Table 3:** Mean absolute error (MAE) and error standard deviation for each frequency range and feature, sorted from the lowest to highest MAE.

Frequency Range	Feature	MAE [RH %]	Error Std [RH %]
Peaks_Region	|Z|	1.847	2.384
Cap_region_Small	|Z|	2.000	2.573
Cap_region_Large	*X*	2.140	2.867
Cap_region_Small	*X*	2.697	3.473
Cap_region_Large	θ	4.900	7.086
Peaks_Region	θ	5.333	7.271
Cap_region_Large	|Z|	8.131	11.735
Cap_region_Large	*R*	10.208	12.644
Cap_region_Small	*R*	11.124	13.480
Full	θ	11.302	14.683
Peaks_Region	*R*	11.716	13.560
Peaks_Region	*X*	12.110	16.200
Full	*R*	12.638	15.020
Cap_region_Small	θ	19.070	20.529
Full	*X*	19.141	21.608
Full	|Z|	19.209	21.809

## Data Availability

This data cannot be made publicly available upon publication as there are no suitable repository for hosting data in this field of study. The data collected in this study are available upon reasonable request.
